# Using More Ecological Paradigms to Investigate Working Memory: Strengths, Limitations and Recommendations

**DOI:** 10.3389/fnhum.2020.00148

**Published:** 2020-05-04

**Authors:** Lison Fanuel, Gaën Plancher, Pascale Piolino

**Affiliations:** ^1^Cognitive Mechanisms Research Laboratory, Université Lyon 2, Bron, France; ^2^Lyon Neuroscience Research Center (CRNL), INSERM U1028, CNRS UMR5292, Université Lyon 1, Université de Lyon, Lyon, France; ^3^Laboratoire Mémoire, Cerveau et Cognition, MC^2^Lab 7536, Université de Paris, Paris, France; ^4^Institut de Psychologie, Université de Paris, Boulogne Billancourt, France; ^5^Institut Universitaire de France, Paris, France

**Keywords:** memory, working memory, virtual reality, naturalistic events, ecological environment

Working memory (WM) is essential to daily-life activities as it allows maintaining information in the short-term while processing concurrent information (Baddeley and Hitch, 1974). For example, one must maintain which ingredient is already in the plate while following a recipe. WM is a complex cognitive function involving multiple processes (e.g., encoding, maintenance, retrieval processes). The present paper focuses on the utility of virtual reality (VR) in investigating maintenance in WM, but the relevance of VR studies also applies to other WM-related mechanisms.

Recent models proposed an attention-based mechanism supporting maintenance of domain-general information: attentional refreshing (or refreshing; Camos et al., 2009; Camos and Barrouillet, 2014; Camos, 2017). Refreshing is described as a brief thought to an information that is no longer perceptually present (Johnson, 1992) and received growing attention in both WM and episodic memory (EM). The WM field provides convincing evidence of an involvement of refreshing in maintenance of visual, spatial, verbal information, as well as in the binding between these information (Hudjetz and Oberauer, 2007; Camos et al., 2009; Vergauwe et al., 2009, 2010, 2012). Studies using delayed recall suggest that memory performance depends on the time available for refreshing (Camos and Portrat, 2015; Souza and Oberauer, 2017; Jarjat et al., 2018) and that refreshing plays a role in construction of episodic traces (Johnson et al., 2002; Loaiza and McCabe, 2013). So far, studies used very simple to-be-remembered material such as letters or spatial locations (e.g., Camos et al., 2009; Vergauwe et al., 2009, 2010; Camos and Portrat, 2015). As refreshing is involved in maintenance of domain-general information and construction of EM, it should play a significant part in maintenance and long-term retention of rich and complex information. Because WM is central in daily-life activities, future research should design more ecological experiments to better understand the role of WM and refreshing in naturalistic situations.

VR seems to be a useful tool to investigate memory functioning in daily-life-like environments. VR allows creating naturalistic situation and increasing their ecological validity as compared to classical experimental or neuropsychological tests (Plancher and Piolino, 2017). Ecological validity refers to the extent to which experimental conditions are similar to a real-world setting (Bohil et al., 2011). Accordingly, a VR experience can provide complex and rich information involving multiple senses (vision, audition, proprioception, etc.) and spatiotemporal features. VR also enables interaction with the environment, for example by controlling displacements, which increase the feeling of immersion in this environment (Mestre and Fuchs, 2006). Besides improving ecological validity, controlled environments can be created to assess multiple features of memory traces—the content of the memory trace (what) and its spatial and temporal location (where and when)—as well as the binding between these features (Plancher et al., 2010). VR is thus a good compromise between memory assessment of daily-life-like experience and experimental control.

While VR was extensively used to better understand EM (Plancher and Piolino, 2017; La Corte et al., 2019), and executive functions (Negut, 2014; Neguţ et al., 2016), only few studies investigated WM mechanisms with this method. Meilinger et al. (2008) investigated the involvement of WM in a wayfinding task. In comparison to a control condition without concurrent processing, a concurrent task (e.g., indicating the spatial location of a sound) negatively affected wayfinding of the routes previously seen. Both verbal and spatial concurrent tasks (continuously repeating a syllable sequence or tapping a spatial sequence, respectively), impaired memory performance for the landmark location and only the spatial concurrent task impaired memory performance for the route (Gras et al., 2013). More recently, Plancher et al. (2018) investigated the role of WM in construction of EM traces using a VR paradigm. While driving into a virtual town, participants had to memorize the encountered scenes as detailed as possible including the elements constituting the scene (*what*), the spatial location (*where*) and the temporal context of the scene (*when*). The recall of the spatial or temporal context associated to each element provided a binding score. As compared to a condition without concurrent processing, a verbal concurrent task (memorizing the number of garbage containers) only impaired memory performance of *what* information and a visuospatial concurrent task (memorizing the spatial position of containers) impaired memory performance of *what, when*, and *what-where-when* binding information. These results suggest that construction of memory traces rely on verbal and visuospatial maintenance mechanisms and were interpreted as reflecting an involvement of both phonological loop (i.e., verbal-specific WM mechanism, Baddeley and Hitch, 1974) and refreshing in the construction of *what* and an involvement of refreshing in the construction of *when* and binding components of EM traces.

Typically, the involvement of refreshing in maintenance is investigated using complex span tasks where to-be-processed items are interleaved in-between each to-be-memorized information (Barrouillet et al., 2004, 2007). Following the assumption that maintenance and processing compete for one limited resource (i.e., attention), increasing the amount of attentional resources required by the processing task leave less attentional resources available for attentional maintenance (i.e., refreshing, Barrouillet et al., 2007). Attentional sharing between maintenance and processing is proposed to rely on time: when time is occupied by a processing task, attentional maintenance cannot take place, and vice versa (Barrouillet et al., 2007). Varying the amount of time required for processing a concurrent task (i.e., its cognitive load) results in manipulating the amount of time available for refreshing. Poorer WM performance under higher cognitive load is taken as evidence of an involvement of refreshing in WM maintenance (Barrouillet et al., 2004, 2007; Vergauwe et al., 2009, 2010).

To understand the involvement of refreshing in maintenance of rich and complex information, we suggest adapting complex span tasks to VR paradigms. The task concurrent to maintenance should be distinct from the memorization task and allow to measure response times. Thereby, it will be possible to manipulate the cognitive load of the concurrent task and investigate whether and how refreshing is involved in maintenance of the different features of a complex memory trace (*what, when, where*) and the binding of these features. A passive exploration of the environment would allow determining the time-course of the task and controlling temporal parameters and require no control nor planning for traveling. Motor and planning actions required by active traveling can constitute an attentional cost (Plancher et al., 2013) and have an uncontrolled detrimental effect on WM performance. However, a passive exploration result in a simple video experience. Active navigation seems more useful to enrich the EM trace (Plancher et al., 2012, 2013; Sauzéon et al., 2012; Jebara et al., 2014). Immersion and real time interaction with the environment are necessary for self-experience and bodily representation and modulate the sense of presence in the present that is central in daily-life experience (Nash et al., 2000; Makowski et al., 2017). Self-experience and bodily representation reinforce EM performance (Bergouignan et al., 2014; Repetto et al., 2016; Tuena et al., 2017, 2019; Blanke et al., 2018) and might also influence maintenance in WM. For a more immersive experience and a better understanding of the involvement of refreshing in daily-life situations, future studies should systematically use an active condition. Contrasting different levels of immersion (from computer screens to head-mounted displays or cave automatic virtual environments) and interaction (from joystick to motion capture) with a passive condition would enable determining the minimum conditions for studying WM in an ecological context and explore how embodiment impacts refreshing.

To study WM in conditions as close as possible from real-life using VR, we suggest designing a virtual environment where the participant is freely exploring and encounter events of different nature (e.g., visual, auditory, proprioceptive, spatial, or any combination). To enhance ecological validity, events should occur at a non-isochronous pace. Temporal parameters related to to-be-memorized and to-be-processed events (number of events, presentation duration, inter-stimuli intervals) should be fixed and participants' behavior (e.g., response times) should be measured. Studies of interdependence between WM processes and other cognitive functions will also benefit from VR paradigms. Long-term memory and semantic representation seem to contribute to refreshing and WM (Loaiza et al., 2015; Loaiza and Camos, 2018). Refreshing is involved in construction of EM traces (Johnson et al., 2002; Loaiza and McCabe, 2013) and might play a part in prospective memory (Marsh and Hicks, 1998). Carefully timed VR experiments manipulating WM parameters will contribute to a detailed insight of these cognitive processes and their relationship with WM.

Developing virtual-reality-based paradigms to investigate WM would be useful to identify the neural basis of WM mechanisms in ecological situations. To date, as for behavioral studies, neurophysiological studies of maintenance in WM (Vogel and Machizawa, 2004; Guimond et al., 2011; Lefebvre et al., 2013; Grimault et al., 2014) and refreshing have used very simple stimuli (Johnson et al., 2005, 2015). To our knowledge, no study combined WM daily-life-like paradigms and neurophysiological measures. Yet, VR paradigms can be combined easily with neurophysiological recording like eye-tracking (e.g., Whitmire et al., 2016) or electrodermal and cardiac responses (e.g., Parsons et al., 2011; Armougum et al., 2019). Electroencephalography (e.g., Jaiswal et al., 2010; Bohil et al., 2011) and fMRI (e.g., Kalpouzos et al., 2010) recordings are possible during exposure in experiments with limited movements. Neurophysiological measures during retrieval depending on WM manipulation during exposure (e.g., the amount of time available for refreshing) could provide a further insight on the implication of WM processes in episodic construction.

VR is a powerful experimental tool that allow creating multimodal and naturalistic environments to assess memory for rich and complex information while keeping a strong experimental control. VR will allow testing theoretical assumptions with enhanced ecological validity and interactive fidelity and explore new hypothesis as whether and how traveling affects WM. In addition, VR allows us to examine some of the basic properties of the situated and embodied approach through exact control of methodological factors and go further into the understanding of the role of presence and consciousness in memory. Yet, using VR paradigms in WM studies require to compromise between a strong control of temporal parameters and immersion through active exploring. We recommend fixing temporal parameters of the experiment and to measure behavior, especially response times, as precisely as possible. Future studies will need to determine the minimum immersion and interaction conditions to explore WM with VR to limit the attentional cost of active navigation and facilitate the combined use of VR and neurophysiological measurements. Moreover, some other technical aspects of VR such as the risk of cybersickness, photorealistic level of environment, type of interaction and way of navigation, level and mode of the embodiment need further investigations to test their impact on WM as suggested by some authors in the domain of memory studies (Smith, 2019).

Future studies should also assess metric properties of VR-based measurements of WM to ensure a good construct validity and reliability. Besides the good equivalence between cognitive performance and physiological responses in the virtual and the real world (Sorita et al., 2013; Armougum et al., 2019), construct validity of VR-based neuropsychological assessments seem suitable (Neguţ et al., 2015), comforting the feasibility of developing VR-based assessment of cognitive functions. Given that VR is becoming more accessible at low cost, future studies will be able to multiply on a larger number of subjects to obtain reliable and reproducible data.

WM is impaired in various populations such as healthy aging, age-related dementia (e.g., Baddeley et al., 1986; Huntley and Howard, 2010) or schizophrenia (e.g., Lee and Park, 2005). In some populations, WM deficit is proposed to be due to an impairment of refreshing (e.g., Hoareau et al., 2016; Fanuel et al., 2018 in healthy aging; Grillon et al., 2013 in schizophrenia). Developing naturalistic tools to investigate WM functioning seem very useful to characterize the WM deficits in these populations. Previous studies suggest that WM training involving multi-modal stimuli, demanding high cognitive engagement and targeting WM domain-general mechanisms are more likely to yield WM and general cognitive enhancement (Morrison and Chein, 2011). VR-based trainings of WM thus seem a promising approach for enhancing both WM and broader cognitive functions. It is a crucial point nowadays to examine the VR acceptability for fragile populations.

## Author Contributions

All authors conceptualized the manuscript. LF wrote the original draft. GP and PP reviewed and edited its intellectual content.

## Conflict of Interest

The authors declare that the research was conducted in the absence of any commercial or financial relationships that could be construed as a potential conflict of interest.

## References

[B1] Armougum A. Orriols E. Gaston-Bellegarde A. Joie-La Marle C. Piolino P. (2019). Virtual reality: a new method to investigate cognitive load during navigation. J. Environ. Psychol. 65:101338 10.1016/j.jenvp.2019.101338

[B2] Baddeley A. D. Hitch G. (1974). Working memory. Psychol. Learn. Motiv. 8, 47–89. 10.1016/S0079-7421(08)60452-19635207

[B3] Baddeley A. D. Logie R. Bressi S. Sala S. D. Spinnler H. (1986). Dementia and working memory. Q. J. Exp. Psychol. Sec. A 38, 603–618. 10.1080/14640748608401616 3809575

[B4] Barrouillet P. Bernardin S. Camos V. (2004). Time constraints and resource sharing in adults' working memory spans. J. Exp. Psychol. Gen. 133, 83–100. 10.1037/0096-3445.133.1.83 14979753

[B5] Barrouillet P. Bernardin S. Portrat S. Vergauwe E. Camos V. (2007). Time and cognitive load in working memory. J. Exp. Psychol. Learn. Mem. Cogn. 33, 570–585. 10.1037/0278-7393.33.3.570 17470006

[B6] Bergouignan L. Nyberg L. Ehrsson H. H. (2014). Out-of-body-induced hippocampal amnesia. Proc. Natl. Acad. Sci. U.S.A. 111, 4421–4426. 10.1073/pnas.1318801111 24616529 PMC3970513

[B7] Bréchet L. Mange R. Herbelin B. Gauthier B. Serino A. Blanke O. (2018). Viewing one's body during encoding boosts episodic memory. bioRxiv [Preprint]. bioRxiv:318956. 10.1101/318956

[B8] Bohil C. J. Alicea B. Biocca F. A. (2011). Virtual reality in neuroscience research and therapy. Nat. Rev. Neurosci. 12, 753–762. 10.1038/nrn3122 22048061

[B9] Camos V. (2017). Domain-specific versus domain-general maintenance in working memory: reconciliation within the time-based resource sharing model. Psychol. Learn. Motiv. 67, 135–171. 10.1016/bs.plm.2017.03.005

[B10] Camos V. Barrouillet P. (2014). Attentional and non-attentional systems in the maintenance of verbal information in working memory: the executive and phonological loops. Front. Hum. Neurosci. 8:900. 10.3389/fnhum.2014.00900 25426049 PMC4224087

[B11] Camos V. Lagner P. Barrouillet P. (2009). Two maintenance mechanisms of verbal information in working memory. J. Mem. Lang. 61, 457–469. 10.1016/j.jml.2009.06.002

[B12] Camos V. Portrat S. (2015). The impact of cognitive load on delayed recall. Psychon. Bull. Rev. 22, 1029–1034. 10.3758/s13423-014-0772-5 25421406

[B13] Fanuel L. Plancher G. Monsaingeon N. Tillmann B. Portrat S. (2018). Temporal dynamics of maintenance in young and old adults. Ann. N. Y. Acad. Sci. 1424, 137–148. 10.1111/nyas.13640 29707786

[B14] Gras D. Gyselinck V. Perrussel M. Orriols E. Piolino P. (2013). The role of working memory components and visuospatial abilities in route learning within a virtual environment. J. Cogn. Psychol. 25, 38–50. 10.1080/20445911.2012.739154

[B15] Grillon M-L. Oppenheim C. Varoquaux G. Charbonneau F. Devauchelle A-.D. Krebs M-.O.. (2013). Hyperfrontality and hypoconnectivity during refreshing in schizophrenia. Psychiatry Res. 211, 226–233. 10.1016/j.pscychresns.2012.09.001 23137808

[B16] Grimault S. Nolden S. Lefebvre C. Vachon F. Hyde K. Peretz I.. (2014). Brain activity is related to individual differences in the number of items stored in auditory short-term memory for pitch: evidence from magnetoencephalography. NeuroImage 94, 96–106. 10.1016/j.neuroimage.2014.03.020 24642285

[B17] Guimond S. Vachon F. Nolden S. Lefebvre C. Grimault S. Jolicoeur P. (2011). Electrophysiological correlates of the maintenance of the representation of pitch objects in acoustic short-term memory: Pitch and ASTM. Psychophysiology 48, 1500–1509. 10.1111/j.1469-8986.2011.01234.x 21824153

[B18] Hoareau V. Lemaire B. Portrat S. Plancher G. (2016). Reconciling two computational models of working memory in aging. Top. Cogn. Sci. 8, 264–278. 10.1111/tops.12184 26748955

[B19] Hudjetz A. Oberauer K. (2007). The effects of processing time and processing rate on forgetting in working memory: testing four models of the complex span paradigm. Mem. Cogn. 35, 1675–1684. 10.3758/BF03193501 18062545

[B20] Huntley J. D. Howard R. J. (2010). Working memory in early Alzheimer's disease: a neuropsychological review. Int. J. Geriatr. Psychiatr. 25, 121–132. 10.1002/gps.2314 19672843

[B21] Jaiswal N. Ray W. Slobounov S. (2010). Encoding of visual–spatial information in working memory requires more cerebral efforts than retrieval: evidence from an EEG and virtual reality study. Brain Res. 1347, 80–89. 10.1016/j.brainres.2010.05.086 20570660 PMC2909367

[B22] Jarjat G. Hoareau V. Plancher G. Hot P. Lemaire B. Portrat S. (2018). What makes working memory traces stable over time?: working memory conditions for long-term recall. Ann. N. Y. Acad. Sci. 1424, 149–160. 10.1111/nyas.13668 29744891

[B23] Jebara N. Orriols E. Zaoui M. Berthoz A. Piolino P. (2014). Effects of enactment in episodic memory: a pilot virtual reality study with young and elderly adults. Front. Aging Neurosci. 6:338. 10.3389/fnagi.2014.00338 25566069 PMC4269133

[B24] Johnson M. K. (1992). MEM: mechanisms of recollection. J. Cogn. Neurosci. 4, 268–280. 10.1162/jocn.1992.4.3.268 23964883

[B25] Johnson M. K. Raye C. L. Mitchell K. J. Greene E. J. Cunningham W. A. Sanislow C. A. (2005). Using fMRI to investigate a component process of reflection: prefrontal correlates of refreshing a just-activated representation. Cogn. Affect. Behav. Neurosci. 5, 339–361. 10.3758/CABN.5.3.339 16396094

[B26] Johnson M. K. Reeder J. A. Raye C. L. Mitchell K. J. (2002). Second thoughts versus second looks: an age-related deficit in reflectively refreshing just-activated information. Psychol. Sci. 13, 64–67. 10.1111/1467-9280.00411 11892780

[B27] Johnson M. R. McCarthy G. Muller K. A. Brudner S. N. Johnson M. K. (2015). Electrophysiological correlates of refreshing: event-related potentials associated with directing reflective attention to face, scene, or word representations. J. Cogn. Neurosci. 27, 1823–1839. 10.1162/jocn_a_00823 25961640 PMC4796063

[B28] Kalpouzos G. Eriksson J. Sjölie D. Molin J. Nyberg L. (2010). Neurocognitive systems related to real-world prospective memory. PLoS ONE 5:e13304. 10.1371/journal.pone.0013304 20949046 PMC2951914

[B29] La Corte V. Sperduti M. Abichou K. Piolino P. (2019). Episodic memory assessment and remediation in normal and pathological aging using virtual reality: a mini review. Front. Psychol. 10:173. 10.3389/fpsyg.2019.00173 30787898 PMC6372520

[B30] Lee J. Park S. (2005). Working memory impairments in schizophrenia: a meta-analysis. J. Abnorm. Psychol. 114, 599–611. 10.1037/0021-843X.114.4.599 16351383

[B31] Lefebvre C. Vachon F. Grimault S. Thibault J. Guimond S. Peretz I.. (2013). Distinct electrophysiological indices of maintenance in auditory and visual short-term memory. Neuropsychologia 51, 2939–2952. 10.1016/j.neuropsychologia.2013.08.003 23938319

[B32] Loaiza V. M. Camos V. (2018). The role of semantic representations in verbal working memory. J. Exp. Psychol. Learn. Mem. Cogn. 44, 863–881. 10.1037/xlm0000475 29648869

[B33] Loaiza V. M. Duperreault K. A. Rhodes M. G. McCabe D. P. (2015). Long-term semantic representations moderate the effect of attentional refreshing on episodic memory. Psychon. Bull. Rev. 22, 274–280. 10.3758/s13423-014-0673-7 24928092

[B34] Loaiza V. M. McCabe D. P. (2013). The influence of aging on attentional refreshing and articulatory rehearsal during working memory on later episodic memory performance. Aging Neuropsychol. Cogn. 20, 471–493. 10.1080/13825585.2012.738289 23116456

[B35] Makowski D. Sperduti M. Nicolas S. Piolino P. (2017). Being there and remembering it: presence improves memory encoding. Consci. Cogn. 53, 194–202. 10.1016/j.concog.2017.06.015 28676191

[B36] Marsh R. L. Hicks J. L. (1998). Event-based prospective memory and executive control of working memory. J. Exp. Psychol. Learn. Mem. Cogn. 24, 336–348. 10.1037/0278-7393.24.2.336 9530843

[B37] Meilinger T. Knauff M. Bülthoff H. H. (2008). Working memory in wayfinding—a dual task experiment in a virtual city. Cogn. Sci. 32, 755–770. 10.1080/03640210802067004 21635352

[B38] Mestre D. Fuchs P. (2006). "Immersion et présence, in *Le traité de la réalité virtuelle* (pp. 309–338). Available online at: http://www.ism.univ-amu.fr/mestre/projects/virtual%20reality/Pres_2005.pdf

[B39] Morrison A. B. Chein J. M. (2011). Does working memory training work? the promise and challenges of enhancing cognition by training working memory. Psychon. Bull. Rev. 18, 46–60. 10.3758/s13423-010-0034-0 21327348

[B40] Nash E. B. Edwards G. W. Thompson J. A. Barfield W. (2000). A review of presence and performance in virtual environments. Int. J. Hum. Comput. Interact. 12, 1–41. 10.1207/S15327590IJHC1201_1

[B41] Neguţ A. Matu S-.A. Sava F. A. David D. (2016). Virtual reality measures in neuropsychological assessment: a meta-analytic review. Clin. Neuropsychol. 30, 165–184. 10.1080/13854046.2016.1144793 26923937

[B42] Negut A. (2014). Cognitive assessment and rehabilitation in virtual reality: theoretical review and practical implications. Roman. J. Appl. Psychol. 16, 1–7. 10.1155/2014/594540

[B43] Neguţ A. Matu S-.A. Sava F. A. David D. (2015). Convergent validity of virtual reality neurocognitive assessment: a meta-analytic approach. Transylv. J. Psychol. 16, 31–54.

[B44] Parsons T. D. Courtney C. G. Arizmendi B. Dawson M. (2011). Virtual Reality Stroop Task for Neurocognitive Assessment. Newport Beach, CA: Medicine Meets Virtual Reality. 21335835

[B45] Plancher G. Barra J. Orriols E. Piolino P. (2013). The influence of action on episodic memory: a virtual reality study. Q. J. Exp. Psychol. 66, 895–909. 10.1080/17470218.2012.722657 23025821

[B46] Plancher G. Gyselinck V. Nicolas S. Piolino P. (2010). Age effect on components of episodic memory and feature binding: a virtual reality study. Neuropsychology 24, 379–390. 10.1037/a0018680 20438215

[B47] Plancher G. Gyselinck V. Piolino P. (2018). The integration of realistic episodic memories relies on different working memory processes: evidence from virtual navigation. Front. Psychol. 9:47. 10.3389/fpsyg.2018.00047 29441037 PMC5797679

[B48] Plancher G. Tirard A. Gyselinck V. Nicolas S. Piolino P. (2012). Using virtual reality to characterize episodic memory profiles in amnestic mild cognitive impairment and Alzheimer's disease: influence of active and passive encoding. Neuropsychologia 50, 592–602. 10.1016/j.neuropsychologia.2011.12.013 22261400

[B49] Plancher G. Piolino P. (2017). Virtual reality for assessment of episodic memory in normal and pathological aging, in The Role of Technology in Clinical Neuropsychology, eds Parsons T. Kane R. (Oxford: Oxford University Press).

[B50] Repetto C. Serino S. Macedonia M. Riva G. (2016). Virtual reality as an embodied tool to enhance episodic memory in elderly. Front. Psychol. 7:1839. 10.3389/fpsyg.2016.01839 27909424 PMC5113123

[B51] Sauzéon H. Arvind Pala P. Larrue F. Wallet G. Déjos M. Zheng X.. (2012). The use of virtual reality for episodic memory assessment: effects of active navigation. Exp. Psychol. 59, 99–108. 10.1027/1618-3169/a000131 22044787

[B52] Smith S. A. (2019). Virtual reality in episodic memory research: a review. Psychon. Bull. Rev. 26, 1213–1237. 10.3758/s13423-019-01605-w 31037605

[B53] Sorita E. N'Kaoua B. Larrue F. Criquillon J. Simion A. Sauzéon H.. (2013). Do patients with traumatic brain injury learn a route in the same way in real and virtual environments? Disabil. Rehabil. 35, 1371–1379. 10.3109/09638288.2012.738761 23244232

[B54] Souza A. S. Oberauer K. (2017). Time to process information in working memory improves episodic memory. J. Mem. Lang. 96, 155–167. 10.1016/j.jml.2017.07.002

[B55] Tuena C. Serino S. Dutriaux L. Riva G. Piolino P. (2019). Virtual enactment effect on memory in young and aged populations: a systematic review. J. Clin. Med. 8:620. 10.3390/jcm8050620 31067784 PMC6572276

[B56] Tuena C. Serino S. Gaston-Bellegarde A. Orriols E. Makowski D. Riva G. (2017). How virtual embodiment affects episodic memory functioning: a proof-of-concept study. Ann. Rev. Cyberther. Telemed. 15, 98–103.

[B57] Vergauwe E. Barrouillet P. Camos V. (2009). Visual and spatial working memory are not that dissociated after all: a time-based resource-sharing account. J. Exp. Psychol. Learn. Mem. Cogn. 35, 1012–1028. 10.1037/a0015859 19586267

[B58] Vergauwe E. Barrouillet P. Camos V. (2010). Do mental processes share a domain-general resource? Psychol. Sci. 21, 384–390. 10.1177/0956797610361340 20424075

[B59] Vergauwe E. Dewaele N. Langerock N. Barrouillet P. (2012). Evidence for a central pool of general resources in working memory. J. Cogn. Psychol. 24, 359–366. 10.1080/20445911.2011.640625

[B60] Vogel E. K. Machizawa M. G. (2004). Neural activity predicts individual differences in visual working memory capacity. Nature 428, 748–751. 10.1038/nature02447 15085132

[B61] Whitmire E. Trutoiu L. Cavin R. Perek D. Scally B. Phillips J. (2016). EyeContact: scleral coil eye tracking for virtual reality, in Proceedings of the 2016 ACM International Symposium on Wearable Computers, 184–191. 10.1145/2971763.2971771

